# Supplementary material for: The interferon-stimulated gene product HERC5 inhibits human LINE-1 retrotransposition with an ISGylation-independent mechanism
Source: Nucleic Acids Res. 2026 Apr 30;54(8):gkag334. doi: 10.1093/nar/gkag334 (PMC13128273; doi:10.1093/nar/gkag334)
Supplement: gkag334_Supplemental_Files [file gkag334_supplemental_files.zip › 260318_supplementary.pdf]

Supplementary information

The interferon-stimulated gene product HERC5 inhibits human LINE-1 retrotransposition with an ISGylation-independent mechanism

Kei Nishimori[1,2], Ahmad Luqman-Fatah[1], Yuzo Watanabe[3], Mari Takahashi[4], Takuhiro Ito[4], Fuyuki Ishikawa[2], and Tomoichiro Miyoshi[1,2,5]

[1] Laboratory for Retrotransposon Dynamics, RIKEN Center for Integrative Medical Sciences, Yokohama 230-0045, Japan

[2] Department of Gene Mechanisms, Graduate School of Biostudies, Kyoto University, Kyoto 606-8501, Japan

[3] Proteomics Facility, Graduate School of Biostudies, Kyoto University, Kyoto 606-8502, Japan

[4] Laboratory for Translation Structural Biology, RIKEN Center for Integrative Medical Sciences, Yokohama 230-0045, Japan

[5] Graduate School of Medical and Dental Sciences, Institute of Science Tokyo, Tokyo 113-8510, Japan

Table S1 List of interacting proteins shared by HERC5 WT and C994A identified by immunoprecipitation-coupled mass spectrometry

Table S2 The actual percentages and relative ratios of EGFP (GFP)-positive cells in this study

Table S3 List of interacting proteins shared by HERC5 and ORF1p identified by immunoprecipitation-coupled mass spectrometry

Figure S1 HERC5 inhibits L1 retrotransposition independently of ISGylation

Figure S2 HERC5 knockdown increases ORF1p levels; RLD deletion alters HERC5 localization

Figure S3 The retrotransposition and protein amount suppression by HERC5 overexpression are ORF1-dependent

Figure S4 HERC5 interacts with L1 RNA and requires full-length ORF1p for protein regulation

Figure S5 HERC5 IP-MS reveals translation-related interactome

Supplementary Figure S1

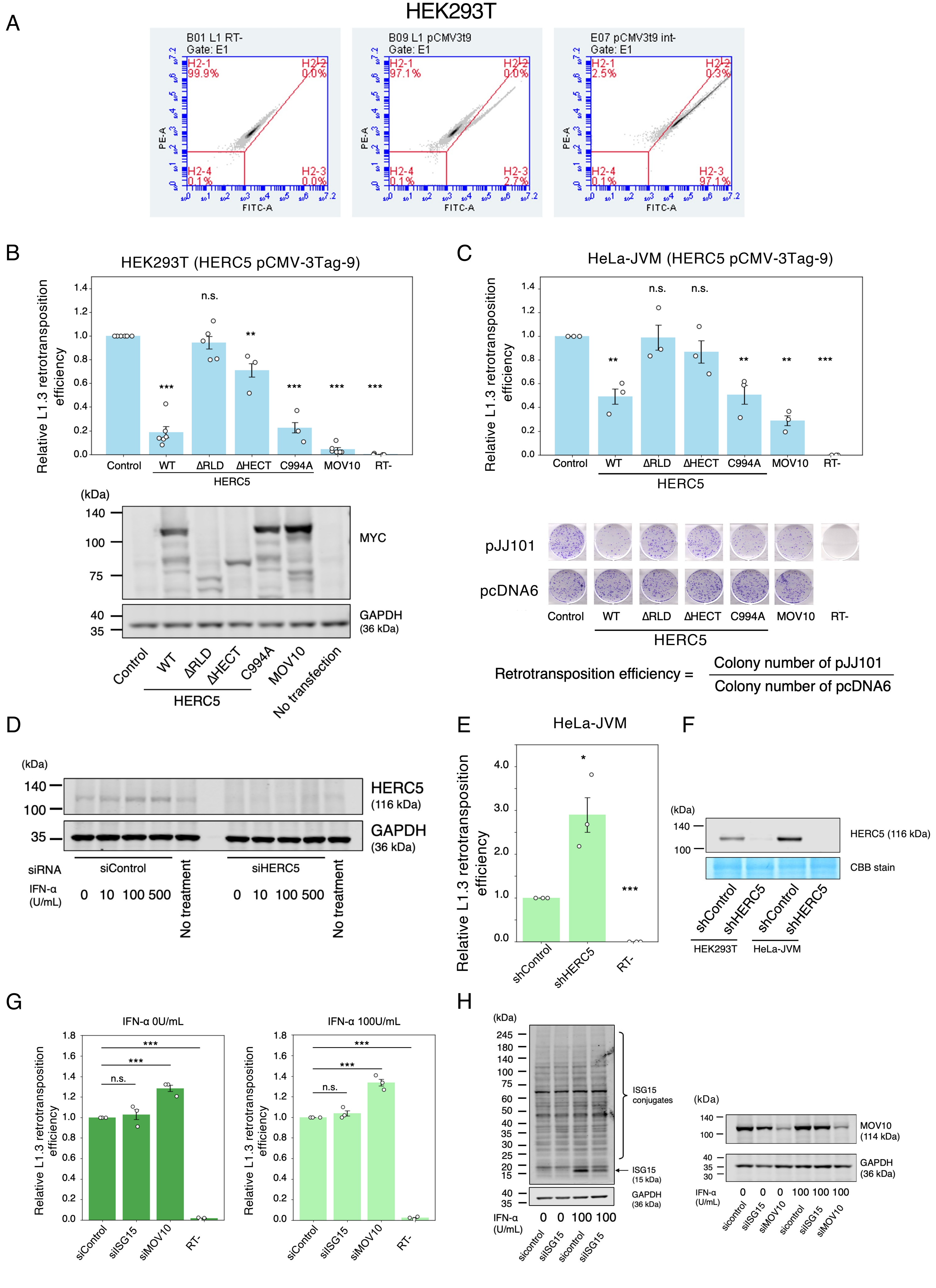

Supplementary Figure S2

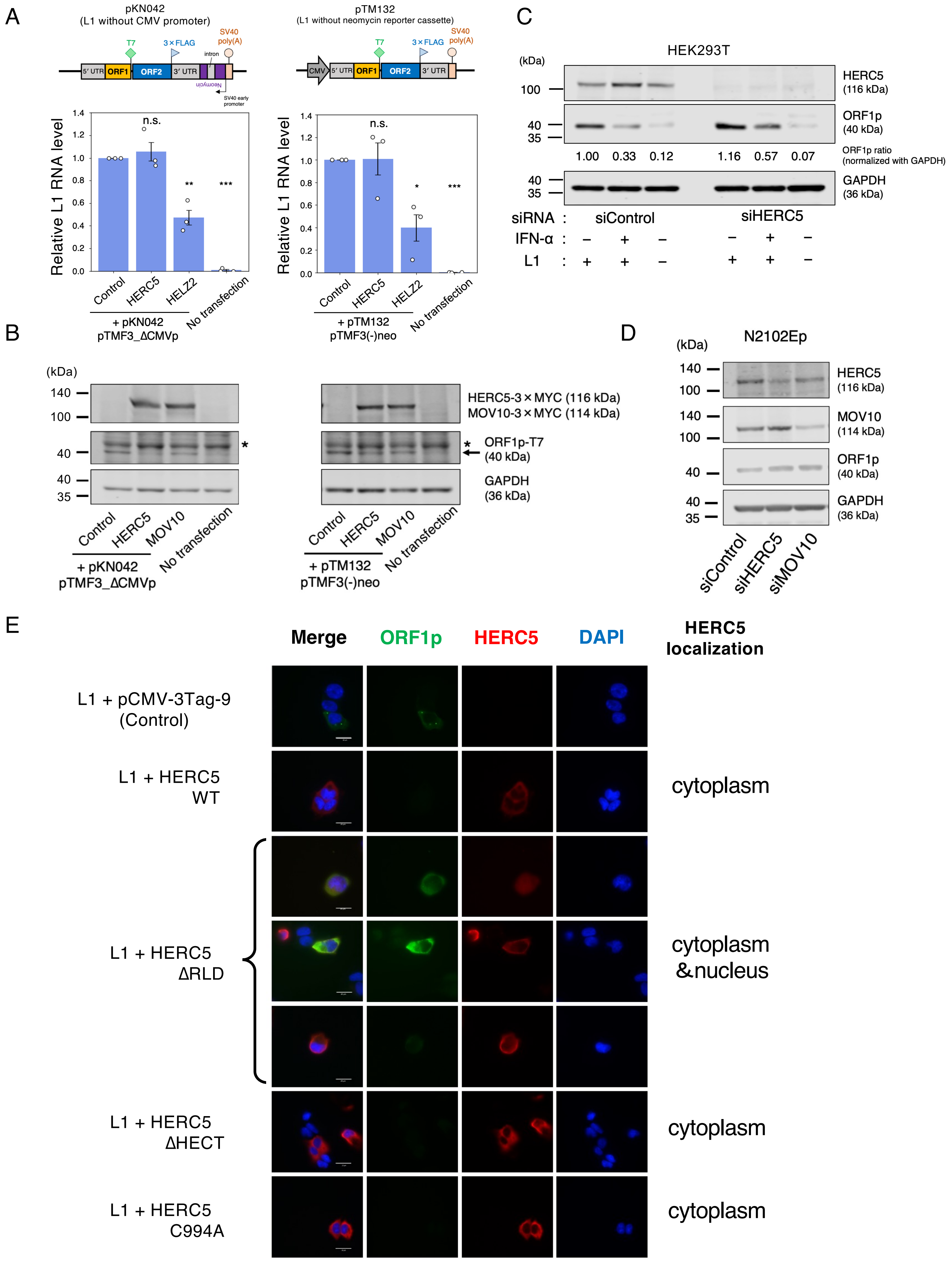

Supplementary Figure S3

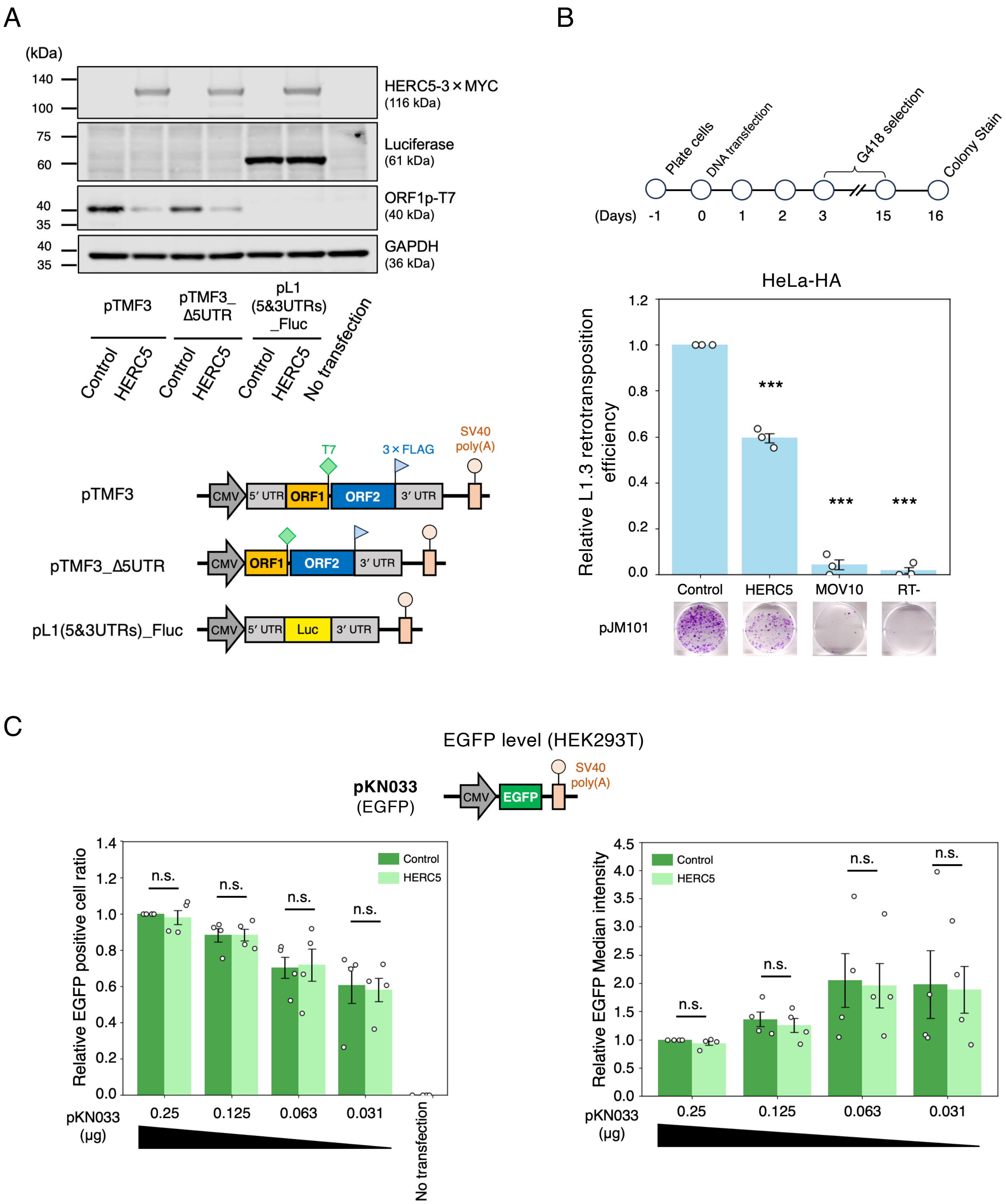

Supplementary Figure S4

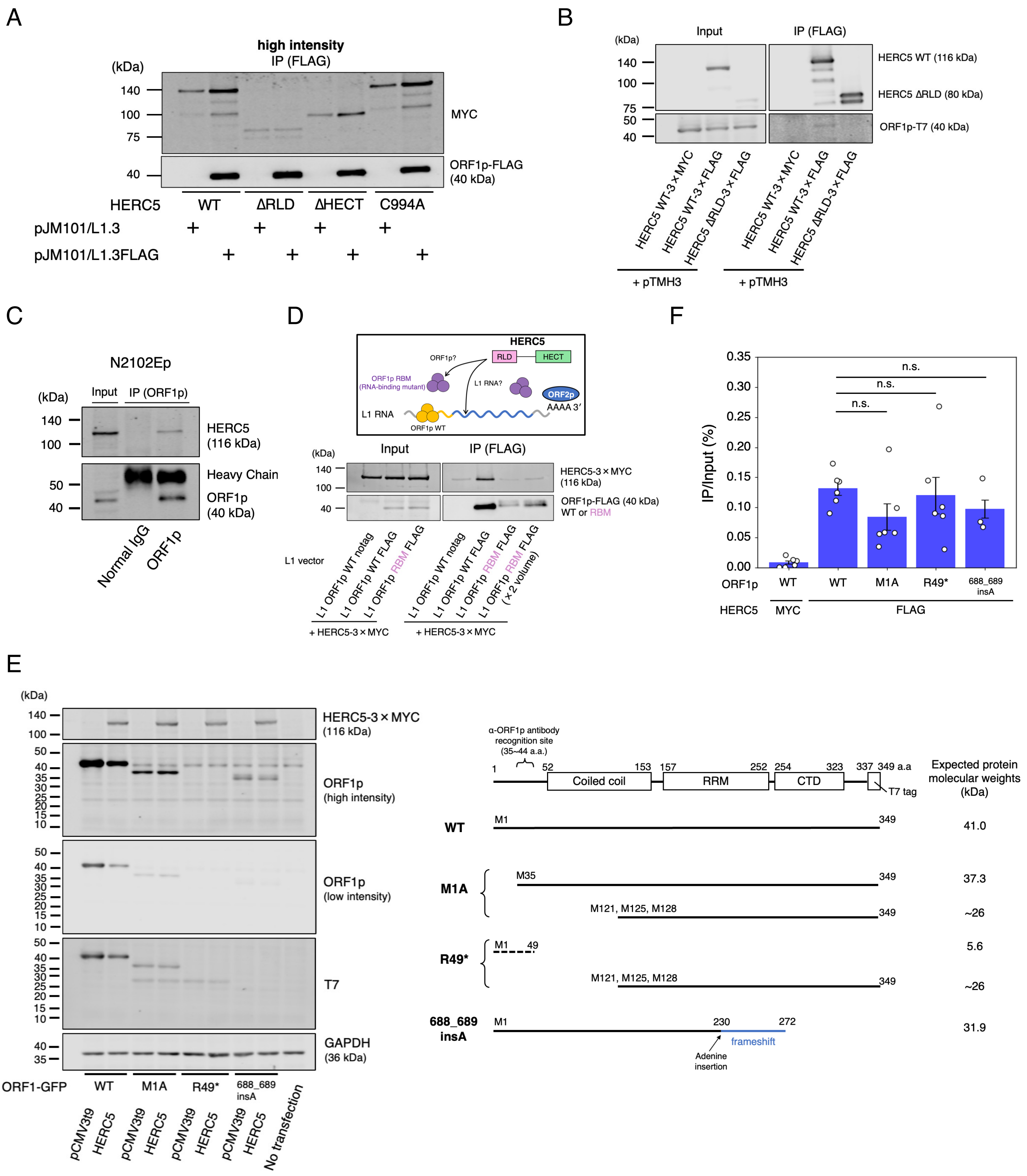

Supplementary Figure S5

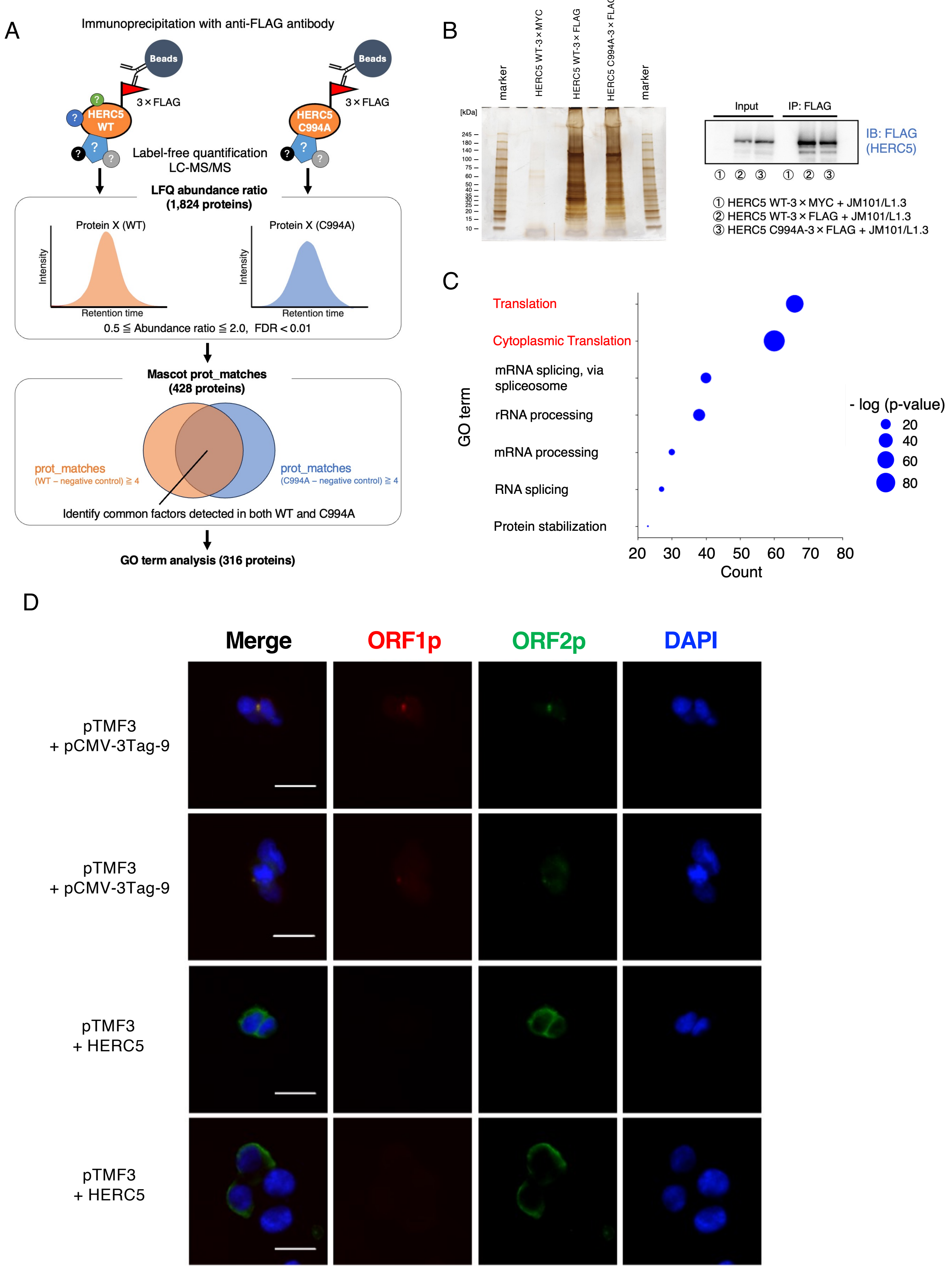

Supplementary Figure S1. HERC5 inhibits L1 retrotransposition independently of ISGylation

(**A**) Representative flow cytometry plots of the L1 retrotransposition assay in HEK293T cells using the *mEGFPI* cassette. Left: the threshold line was set based on the scatter plot of cepB-gfp-L1.3RT(-), which served as the negative control. X-axis, fluorescein isothiocyanate-area (FITC-A). Y-axis, phycoerythrin-area (PE-A). Middle: the cells in the H2-3 gate were determined to be EGFP-positive. Right: the percentage of EGFP-positive cells from the intronless EGFP reporter cassette (transfection control). (**B**) L1 retrotransposition assay in HEK293T cells using the pCMV-3Tag-9 vector system. Top: L1 retrotransposition assay with HERC5 overexpression using pCMV-3Tag-9 in HEK293T. MOV10 and RT-deficient L1 (cepB-gfp-L1.3RT[-]) served as controls. X-axis, name of the transfected constructs. Y-axis, relative L1 retrotransposition efficiency compared to the control (pCMV-3Tag-9, set to 1.0). The error bars represent the mean ± the standard error of the mean (SEM) of at least three independent biological replicates. Each dot represents an independent biological replicate. The *p*-values were calculated using a one-way ANOVA followed by Bonferroni-Holm post-hoc tests; * $p < 0.05$, ** $p < 0.01$, *** $p < 0.001$; n.s.: not significant. Bottom: Protein expression levels of HERC5 WT and mutants in HEK293T. Cells were transfected with HERC5 constructs (pCMV-3Tag-9 vector system). HERC5 and GAPDH proteins were detected by western blot using anti-MYC and anti-GAPDH antibodies, respectively. GAPDH served as a loading control. (**C**) L1 retrotransposition assay in HeLa-JVM using the pCMV-3Tag-9 vector system. Top: HeLa-JVM were co-transfected with an L1 expression construct with an *mblastI* retrotransposition indicator cassette (pJJ101) and either HERC5 WT, ∆RLD, ∆HECT, C994A, or MOV10. Cells were selected with blasticidin (10 µg/mL), stained with crystal violet, and the resulting colonies were counted. MOV10 and the RT mutant served as controls. X-axis, name of the transfected constructs. Y-axis, relative L1 retrotransposition efficiency compared to the control (pCMV-3Tag-9, set to 1.0). The error bars and *p*-values were calculated as in (B). Bottom: representative images of stained blasticidin-resistant colonies. The colony numbers of pJJ101 were normalized to those of pcDNA6 to determine retrotransposition efficiency. (**D**) Endogenous HERC5 protein expression levels with different concentrations of IFN-α (0, 10, 100, and 500 U/mL) and siRNA treatments in HEK293T cells. HERC5 and GAPDH proteins were detected by western blot using anti-HERC5 and anti-GAPDH antibodies, respectively. GAPDH served as a loading control. (**E**) L1 retrotransposition assay with HERC5 knockdown. HeLa-JVM cells were transfected with the WT L1-expressing construct (cepB-gfp-L1.3). The RT-deficient L1 (cepB-gfp-

L1.3RT[-]) served as a negative control. After blasticidin selection (10 μg/mL), the percentage of EGFP-positive cells was measured. X-axis, name of the cell line (shControl or shHERC5) or the RT(-) mutant. Y-axis, relative L1 retrotransposition efficiency compared to the control knockdown (set to 1.0). The error bars and *p*-values were calculated as in (B). (**F**) Endogenous HERC5 expression levels in the respective shRNA-knockdown cells. An equal amount of total protein was subjected to western blot analysis. HERC5 was detected using an anti-HERC5 antibody. The membrane was stained with CBB Stain One (Ready To Use) (Nacalai Tesque) and served as a loading control. (**G**) L1 retrotransposition assay with IFN-α and ISG15 knockdown in HEK293T cells. HEK293T cells were treated with IFN-α (100 U/mL) (day −2) and siRNA (day −1) and then transfected with the WT L1-expressing construct (cepB-gfp-L1.3) (day 0). The RT-deficient L1 (cepB-gfp-L1.3RT[-]) served as a negative control, and siMOV10 served as a positive control. After blasticidin selection (10 μg/mL), the percentage of EGFP-positive cells was measured. Left: the relative L1 retrotransposition assay with IFN-α 0 U/mL and siRNA treatments in HEK293T cells. Right: the relative L1 retrotransposition assay with IFN-α 100 U/mL and siRNA treatments in HEK293T cells. X-axis, siRNA treatment. Y-axis, relative L1 retrotransposition efficiency to each non-targeting siControl (set to 1.0). The error bars and *p*-values were calculated as in (B). Each dot represents an independent biological replicate. (**H**) Endogenous ISG15 and MOV10 expression levels with IFN-α and siRNA treatments in HEK293T cells. HERC5, MOV10, and GAPDH were detected by western blot using anti-HERC5, anti-MOV10, and anti-GAPDH antibodies, respectively. GAPDH served as a loading control. Upon IFN-α treatment, free ISG15 was detected at approximately 15 kDa.

Supplementary Figure S2. HERC5 knockdown increases ORF1p levels; RLD deletion alters HERC5 localization

(**A**) L1 RNA levels from pKN042 and pTM132. Top: schematic of the two L1 constructs. pKN042 expresses L1 without CMV promoter, and pTM132 expresses L1 without neomycin reporter cassette in the 3′ UTR. Bottom: Relative L1 RNA levels with HERC5 overexpression. The T7 primer pair was used to quantify L1 RNA levels, which were normalized to GAPDH RNA levels. X-axis, name of the transfected constructs. Y-axis, relative L1 RNA level compared to each control (pCMV-3Tag-9, set to 1.0). The error bars represent the mean ± the standard error of the mean (SEM) of three independent biological replicates. Each dot represents an independent biological replicate. The *p*-values were calculated using a one-way ANOVA followed by Bonferroni-Holm post-hoc tests; * $p < 0.05$, ** $p < 0.01$, *** $p < 0.001$; n.s.: not significant. (**B**) ORF1p levels from pKN042 and pTM132 with HERC5 overexpression. HERC5 and MOV10 were detected by an anti-MYC antibody. ORF1p and GAPDH were detected by anti-T7 and anti-GAPDH antibodies, respectively. GAPDH served as a loading control. The asterisk (*) in the membrane stained with anti-T7 antibody indicates non-specific signal. (**C**) ORF1p levels with siRNA and IFN-α treatments. HEK293T cells were treated with 100 U/mL of IFN-α (day −2) and siRNA (day −1) and then transfected with the WT L1-expressing plasmid (pJM101/L1.3). HERC5, ORF1p, and GAPDH were detected using anti-HERC5, anti-ORF1p, and anti-GAPDH antibodies, respectively. GAPDH served as a loading control. ORF1p signal intensities were quantified using Empiria Studio and normalized to GAPDH to obtain the ORF1p/GAPDH ratio. (**D**) ORF1p levels with siRNA treatment in the N2102Ep cell line. N2102Ep cells were treated with siRNA and protein levels were assessed by western blotting. HERC5, MOV10, ORF1p, and GAPDH were detected using anti-HERC5, anti-MOV10, anti-ORF1p, and anti-GAPDH antibodies, respectively. GAPDH served as a loading control, and siMOV10 served as a positive control. (**E**) Representative immunofluorescence images showing the localization of HERC5 WT, its mutants, and ORF1p. HEK293T cells were co-transfected with pJM101/L1.3FLAG and either the empty vector, HERC5 WT, or the indicated mutants. The localization pattern of HERC5 is noted in the rightmost column. ORF1p was detected in green, HERC5 and its mutants in red, and nuclei were counterstained with DAPI (blue). Scale bar, 25 μm.

Supplementary Figure S3. The retrotransposition and protein amount suppression by HERC5 overexpression are ORF1-dependent

(**A**) ORF1p and ORF2p levels with HERC5. Top: HEK293T cells were co-transfected with the modified L1 constructs and a HERC5-expressing vector. The cells were harvested 3 days post-transfection. HERC5, luciferase, ORF1p, and GAPDH were detected by anti-MYC, anti-luciferase, anti-T7, and anti-GAPDH antibodies, respectively. GAPDH served as a loading control. Bottom: schematic of modified L1 vectors. pTMF3 expresses ORF1p tagged with a T7 gene 10 epitope and ORF2p tagged with a 3×FLAG epitope at their carboxyl termini. pTMF3_Δ5UTR is a derivative of pTMF3 that does not contain the L1 5′ UTR sequence. pL1 (5&3UTRs)_Fluc is a derivative of pTMF3 that contains the firefly luciferase gene in place of the L1.3 coding sequence. (**B**) L1 retrotransposition assay in HeLa-HA. Top: timeline of the assay. HeLa-HA cells were co-transfected with an L1-expressing construct (pJM101) and either pCMV-3Tag-9, HERC5, or MOV10. Cells were selected with G418 (500 μg/mL), stained with crystal violet, and the resulting colonies were counted. The representative images of stained G418-resistant colonies are shown below each condition. The colony numbers of pJM101 were normalized to transfection efficiency to determine retrotransposition efficiency. MOV10 and the RT mutant (pJM105) served as controls. X-axis, name of the transfected constructs. Y-axis, relative L1 retrotransposition efficiency compared to the control (pCMV-3Tag-9, set to 1.0). The error bars represent the mean ± the standard error of the mean (SEM) of three independent biological replicates. Each dot represents an independent biological replicate. The *p*-values were calculated using a one-way ANOVA followed by Bonferroni-Holm post-hoc tests; *** $p < 0.001$. (**C**) Flow cytometry analysis measuring EGFP expression with various plasmid amounts. HEK293T cells were co-transfected with various amounts of pKN033 (0.25, 0.125, 0.063, and 0.031 μg) and either pCMV-3Tag-9 or HERC5. Top: schematic of the pKN033 plasmid. Bottom: relative ratio (left) and median intensities (right) of EGFP-positive cells, respectively. X-axis, the amounts of pKN033. Y-axis, relative ratio or median intensities compared to the control (pCMV-3Tag-9, set to 1.0). The error bars represent the mean ± the standard error of the mean (SEM) of four independent biological replicates. Each dot represents an independent biological replicate. The *p*-values were calculated using a two-tailed, unpaired Student's t-test; n.s.: not significant.

Supplementary Figure S4. HERC5 interacts with L1 RNA and requires full-length ORF1p for protein suppression

(**A**) Interaction of ORF1p with HERC5 WT or its mutants. The MYC blot shows the high-intensity image of Figure 4A. The ORF1p-FLAG blot is identical to that shown in Figure 4A. HERC5 and its mutants were detected by an anti-MYC antibody, and ORF1p was detected by an anti-FLAG antibody. (**B**) Interaction of HERC5-FLAG with ORF1p-T7. HEK293T cells were co-transfected with L1 (pTMH3) and HERC5 WT or ΔRLD expression vectors. Cells were harvested 4 days post-transfection, and HERC5-3×FLAG complexes were immunoprecipitated. HERC5 was detected by an anti-MYC antibody, and ORF1p-T7 was detected by an anti-T7 antibody. (**C**) Interaction of the endogenous ORF1p with HERC5 in N2102Ep cells. ORF1p-complexes were immunoprecipitated from N2102Ep cells. HERC5 and ORF1p were detected by anti-HERC5 and anti-ORF1p antibodies, respectively. (**D**) Interaction of the ORF1p RBM with HERC5. HEK293T cells were co-transfected with HERC5 and either L1 WT or RBM expression vectors. Top: schematic diagram of L1 RNP with ORF1p WT (yellow) and RBM (purple). Bottom: the input and anti-FLAG IP reactions were analyzed by western blotting. FLAG-tagged ORF1p WT and RBM were immunoprecipitated. HERC5 and ORF1p were detected by anti-MYC and anti-FLAG antibodies, respectively. (**E**) L1 mutants' protein expression analysis. Left: To assess whether the start codon mutant (M1A), the premature stop codon mutant (R49*), or the frameshift mutant (688_689insA) produce the proteins, we performed the western blotting using antibodies against ORF1p (recognizes N-terminal 35–44 amino acids of ORF1p) and the T7 epitope tag at the C-terminus, so that only proteins translated in the same reading frame as WT ORF1p are detectable by western blot. HERC5 and GAPDH were detected by anti-MYC and anti-GAPDH antibodies, respectively. Right: the schematic diagram of expressed proteins from mutant L1 and their expected molecular weights, including a C-terminus epitope tag. ORF1p WT produced 41 kDa proteins, which were detected by both anti-ORF1p and anti-T7 antibodies. The M1A mutant produced two distinct products: one predicted to be initiated at the second methionine in ORF1 (M35), and another seems to be initiated at M120, M125, or M128 (hereafter referred to as fourth methionine cluster in *ORF1*), considering their molecular weight, ~26 kDa. The product from M35 was detected by both anti-ORF1p and anti-T7 antibodies (upper band of T7 antibody signal, molecular weight is expected to be 37.3 kDa). The R49* mutant produced a detectable protein whose translation seems to be initiated from the fourth methionine cluster, which was also detected from the M1A mutant. In contrast, the translated product corresponding to M1–R49 (48 amino acids in length, its

molecular weight is expected to be 5.6 kDa) was likely too small to be detected by western blot. The 688_689insA mutant harbors an adenine insertion between nucleotides 688 and 689, which induces a frameshift downstream of the insertion and introduces a premature stop codon at the 272 amino acids. This 688_689insA mutant is predicted to produce a protein of ~32 kDa, which corresponds to the detected signal by anti-ORF1p antibody.

(**F**) RNA-IP experiment to measure HERC5 and ORF1 mutants RNA interactions. HEK293T cells were co-transfected with an ORF1 mutant and HERC5-expressing vectors. Cells were harvested 4 days post-transfection, and HERC5-3×FLAG complexes were immunoprecipitated. After the immunoprecipitation, co-immunoprecipitated L1 RNA amounts were measured by RT-qPCR, and the ratio of anti-FLAG IP fractions/Input was calculated. The error bars represent the mean ± the standard error of the mean (SEM) of more than three independent biological replicates. Each dot represents an independent biological replicate. The *p*-values were calculated using a one-way ANOVA followed by Bonferroni-Holm post-hoc tests; n.s.: not significant.

Supplementary Figure S5. HERC5 IP-MS reveals translation-related interactome

(**A**) Rationale for HERC5 WT and C994A immunoprecipitation-coupled with mass spectrometry. HEK293T cells were co-transfected with an L1-expressing plasmid (pJM101/L1.3) and either HERC5 WT-3×FLAG, HERC5 C994A-3×FLAG, or HERC5 WT-3×MYC (negative control). Cells were harvested 4 days post-transfection, and HERC5-3×FLAG complexes were purified using an anti-FLAG antibody. Proteins interacting with HERC5 WT and C994A were analyzed by LC–MS/MS, and label-free quantification (LFQ) was performed to determine the relative abundance of proteins. A total of 1,824 proteins were selected based on LFQ abundance ratios and FDR confidence scores, and 428 proteins were selected based on Mascot prot_matches. Gene Ontology (GO) term analysis was performed on the retained 316 proteins common to both filters (abundance ratio and prot_matches criterion) as candidate interactors of HERC5 WT and C994A. (**B**) Silver-stained gel image of proteins co-immunoprecipitated with HERC5. Left: HERC5 WT and C994A complexes purified by immunoprecipitation were separated by SDS-PAGE and visualized by silver staining. The gel was excised into 18 slices according to molecular weight and subjected to mass spectrometry. Right: western blot analysis confirming HERC5 WT and C994A immunoprecipitation. HERC5 was detected using an anti-FLAG antibody. (**C**) Enriched GO terms based on the biological process (BP) among the selected proteins. X-axis, protein count (number of proteins identified by mass spectrometry). Y-axis, name of GO BP terms. Circle size, $-\log_{10}$ (*p*-value). The top enriched GO terms are shown. (**D**) Representative immunofluorescence images showing the localization of ORF1p and ORF2p. HEK293T cells were co-transfected with L1-expressing plasmid (pTMF3) and either the empty vector or HERC5-expressing vector. ORF1p and ORF2p were detected in red and green, respectively. The nuclei were counterstained with DAPI (blue). Scale bar, 25 μm.
